# *Agrobacterium tumefaciens* mediated transformation of the aquatic carnivorous plant *Utricularia gibba*

**DOI:** 10.1186/s13007-020-00592-7

**Published:** 2020-04-10

**Authors:** A. Oropeza-Aburto, S. A. Cervantes-Pérez, V. A. Albert, L. Herrera-Estrella

**Affiliations:** 1 Laboratorio Nacional de Genómica para la Biodiversidad, Centro de Investigación y de Estudios Avanzados del Instituto Politécnico Nacional, 36824 Irapuato, Guanajuato Mexico; 2 Department of Biological Sciences, University at Buffalo, Buffalo, NY 14260 USA; 3 School of Biological Sciences, Nanyang Technological University, Singapore, 637551 Singapore; 4 Institute of Genomics for Crop Abiotic Stress Tolerance, Plant and Soil Department, Texas Tech University, Lubbock, USA

**Keywords:** *Agrobacterium tumefaciens*, Genetic transformation, Carnivorous plant

## Abstract

**Background:**

The genus *Utricularia* belongs to Lentibulariaceae, the largest family of carnivorous plants, which includes terrestrial, epiphytic and aquatic species. The development of specialized structures that evolved for carnivory is a feature of this genus that has been of great interest to biologists since Darwin‘s early studies. *Utricularia gibba* is itself an aquatic plant with sophisticated bladder traps having one of the most complex suction mechanisms for trapping prey. However, the molecular characterization of the mechanisms that regulate trap development and the biophysical processes involved in prey trapping are still largely unknown due to the lack of a simple and reproducible gene transfer system.

**Results:**

Here, we report the establishment of a simple, fast and reproducible protocol for genetic transformation of *U. gibba* based on the T-DNA of *Agrobacterium tumefaciens*. An in vitro selection system using Phosphinotricin as a selective agent was established for *U. gibba*. Plant transformation was confirmed by histochemical GUS assays and PCR and qRT-PCR analyses. We report on the expression pattern of the 35S promoter and of the promoter of a trap-specific ribonuclease gene in transgenic *U. gibba* plants.

**Conclusions:**

The genetic transformation protocol reported here is an effective method for studying developmental biology and functional genomics of this genus of carnivorous plants and advances the utility of *U. gibba* as a model system to study developmental processes involved in trap formation.

## Background

Dependence on animal prey is not restricted to the animal kingdom; among the angiosperms, carnivorous plants have been reported since Darwin’s early works [1, 2]. Darwin was fascinated by the rapid prey capturing movements of many carnivorous plants, which he called “*the most wonderful plants in the world*” (C. Darwin, *Insectivorous plants*, p. 231) [1]. This complex trait evolved independently at least six times across flowering plant phylogeny [3, 4], where carnivorous plants are represented in five different plant orders: Poales (monocots), Caryophyllales (core eudicots), Oxalidales (rosids), Ericales and Lamiales (asterids). The extent of morphological and physiological adaptations to capture prey is extensive across taxa, involving features associated with attraction, retention, trapping and digestion of animals and absorption of nutrients from their breakdown products [5–7]. These remarkable features undoubtedly lead to an increase in fitness for species that engage in this lifestyle. However, the development of specialized structures, attractants and secretions ensuring both prey attraction and capture [8–11] appears to be costly for the plant in both energetic and metabolic terms [4].

Among carnivorous plants, the largest (around 220 species) and one of the most cosmopolitan genera is *Utricularia*, which includes terrestrial, epiphytic and aquatic species [12]. The traps, which are characteristic of the genus, are complex structures usually 1 to 6 mm long [13] that employ a complex suction mechanism to capture and digest small invertebrates [14]. Inside the bladder traps, there are two type of glands. The bifid glands, located at the entrance of the trap, are responsible for expelling water to generate negative pressure for prey suction [13]. The quadrifid glands are involved in secretion of digestive enzymes that cover almost the entire inner trap surface [15–18]. There are few reports about digestion mechanisms inside the traps or the enzymes involved in the process. However, the presence of proteases, esterases and acid phosphatases has been reported [19–22]. Phosphatases occurring at different subcellular levels have been found in several families of carnivorous plants. In *Utricularia*, quadrifid glands play an important role in phosphatase secretion for hydrolysis of organic phosphorus compounds [20, 23, 24]. It has also been suggested that zooplankton communities and microorganisms inside the trap could also play an important role in the process of prey digestion [24, 25].

The carnivorous lifestyle has undergone detailed study to understand genetic mechanisms underlying the habit [26, 27], to discover digestive enzymes [22, 28], and to explore the structure and properties of traps during prey capture [9, 29, 30]. Regarding molecular research, genome size studies revealed that some carnivorous plants have the smallest genomes among angiosperms. Among them, *Utricularia gibba* (ca. 100 Mbp) [31] has the smallest known plant genome that has been corroborated by third-generation whole genome sequencing [32, 33].

Genetic transformation is a fundamental tool to study gene structure and function in plants. Among plant transformation systems, the Ti plasmid of *Agrobacterium tumefaciens* has been shown to be the most versatile and efficient for many different plant species. However, in carnivorous plant species, genetic transformation has so far been limited. *Agrobacterium*-mediated transformation of the carnivorous medicinal plant *Drosera rotundifolia* has been reported and used to alter naphthoquinone content [34–36]. It would be advantageous to establish transformation systems for other carnivorous plant species to facilitate refined genetic studies of their metabolism, development and evolution. Here we report the establishment of a simple, fast and reproducible transformation method for *Utricularia gibba*. This *Agrobacterium*-based system allowed us to identify a trap-specific promoter. Our work should allow the development of various molecular tools, such as overexpressing lines, RNAi lines and CRISPR/Cas9 knockout mutants [37], to study genes involved in development and differentiation of organs, particularly those involved in the development and functionality of bladder traps.

## Results

### Selectable marker for *U. gibba* transformation protocol

To identify an appropriate resistance gene for *U. gibba* transformation, we tested the effect of different concentrations of selective agents previously used for developing plant transformation systems, such as Hygromycin (Hyg, aminocyclitol antibiotic) [38], Kanamycin (Kan, aminoglycoside antibiotic) [39] and Glufosinate-ammonium PESTANAL (PPT) on the growth of *U. gibba* plants. Hyg and Kan inhibit protein synthesis, whereas PPT is an herbicide that inhibits glutamine synthetase, causing accumulation of toxic levels of ammonia in the cells [40]. *U. gibba* plants were grown for two weeks in liquid media containing different concentrations of these selective agents. *U. gibba* plants cultivated with 20, 40, 60 and 80 mg/l of Kanamycin survived all concentrations during their growth kinetics (See Additional file 1: Figure S1). Tissues treated with 5, 10, 20 and 30 mg/l Hygromycin showed a slight yellowing and sporadic dead tissue. However, even at the highest concentration of Hygromycin, most tissue remained green and viable (See Additional file 1: Figure S1). By contrast, after 9 days of exposure to all concentrations of PPT (6, 10, 15 and 20 mg/l), plants showed gradual whitening, and after 2 weeks, all tissues were dead. We selected 10 mg/l of PPT to supplement selective media for all subsequent transformation experiments (See Additional file 1: Figure S1).

### Transformation protocol

**Fig. 1:**
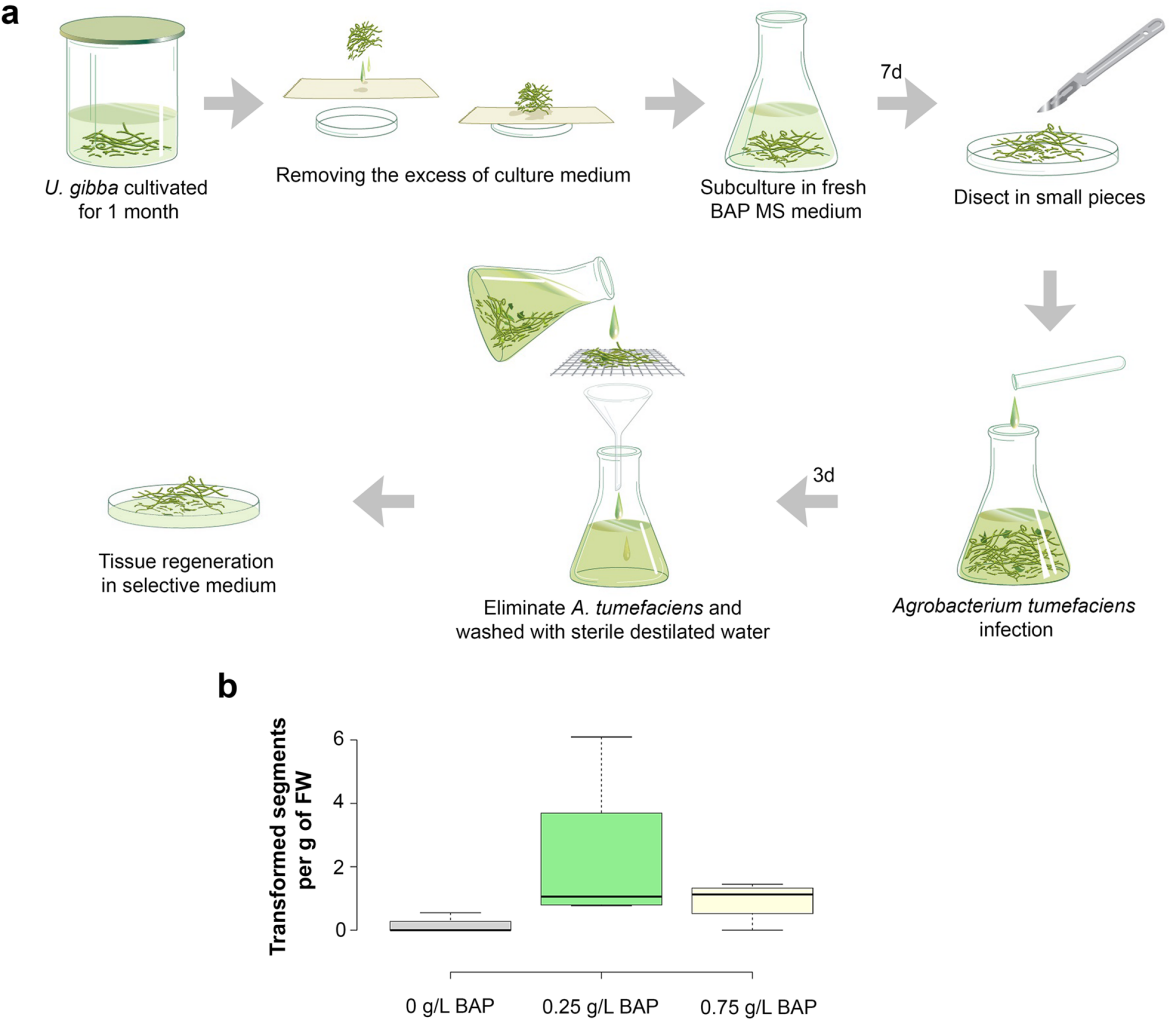
Protocol and transformation efficiency. **a** Diagram illustrating the protocol used for *U. gibba* transformation. *U. gibba* tissue, propagated for 1 month in liquid media, was collected and placed on absorbent paper to remove excess liquid. Collected tissue was grown for an additional week in fresh MS media containing BAP and then cut into small pieces with a scalpel. The segments of *U. gibba* tissue were transferred to MS with BAP and inoculated with an *Agrobacterium* culture overnight for co-cultivation. After 3 days of co-culture in continuous agitation, *Agrobacterium* was removed by filtering *U. gibba* tissue through a plastic mesh, followed by sterile water washes. Clean *U. gibba* tissue was placed in selective medium containing an antibiotic to kill remaining bacterial cells. **b** Box plot showing the number of transformants per gram of fresh weight carrying the p35S-GUS::GFP construct that were obtained when tissue was exposed to different concentrations of BAP. The results represent the means and standard deviations of four independent experiments

To determine whether the cytokinin benzyl amino purine (BAP) could induce a greater number of transformants by increasing de novo shoot formation, *U. gibba* explants were grown for a week in MS medium supplemented with 0, 0.25 or 0.75 mg/l of BAP prior to co-cultivation. Explants were then co-cultivated for 72 h with *Agrobacterium* strain GV2260 carrying a binary vector containing the p35S-GUS::GFP construct and the bialaphos resistance gene (bar) as a selectable marker for resistance to PPT. They were then transferred to PPT selective media for 2 weeks, each explant returning to BAP concentrations as previously described. All treatments were then transferred to selective media without BAP. Four independent biological replicates were used for each treatment. For explants incubated in media without BAP, we obtained a transformation efficiency of 0.14 transformants per gram of tissue, 0.9 for explants incubated with 0.75 mg/l of BAP, and 2.24 for media containing 0.25 mg/l BAP. Although an ANOVA test showed that there was not a significant difference between the BAP treatments, we found reproducibly higher efficiencies using 0.25 mg/l BAP in the selective media (Fig. 1b). The selection procedure was very effective, as no escape events were detected. All plantlets rescued after transformation with the p35S-GUS::GFP construct showed homogeneous GUS staining and were PCR positive (Fig. 3; Additional file 1: Figure S4). Moreover, the results were reproducible using the protocol with 0.25 mg/l BAP and a vector carrying a reporter gene under the control of the promoter of a previously reported gene with trap-specific expression [41].

**Fig. 2:**
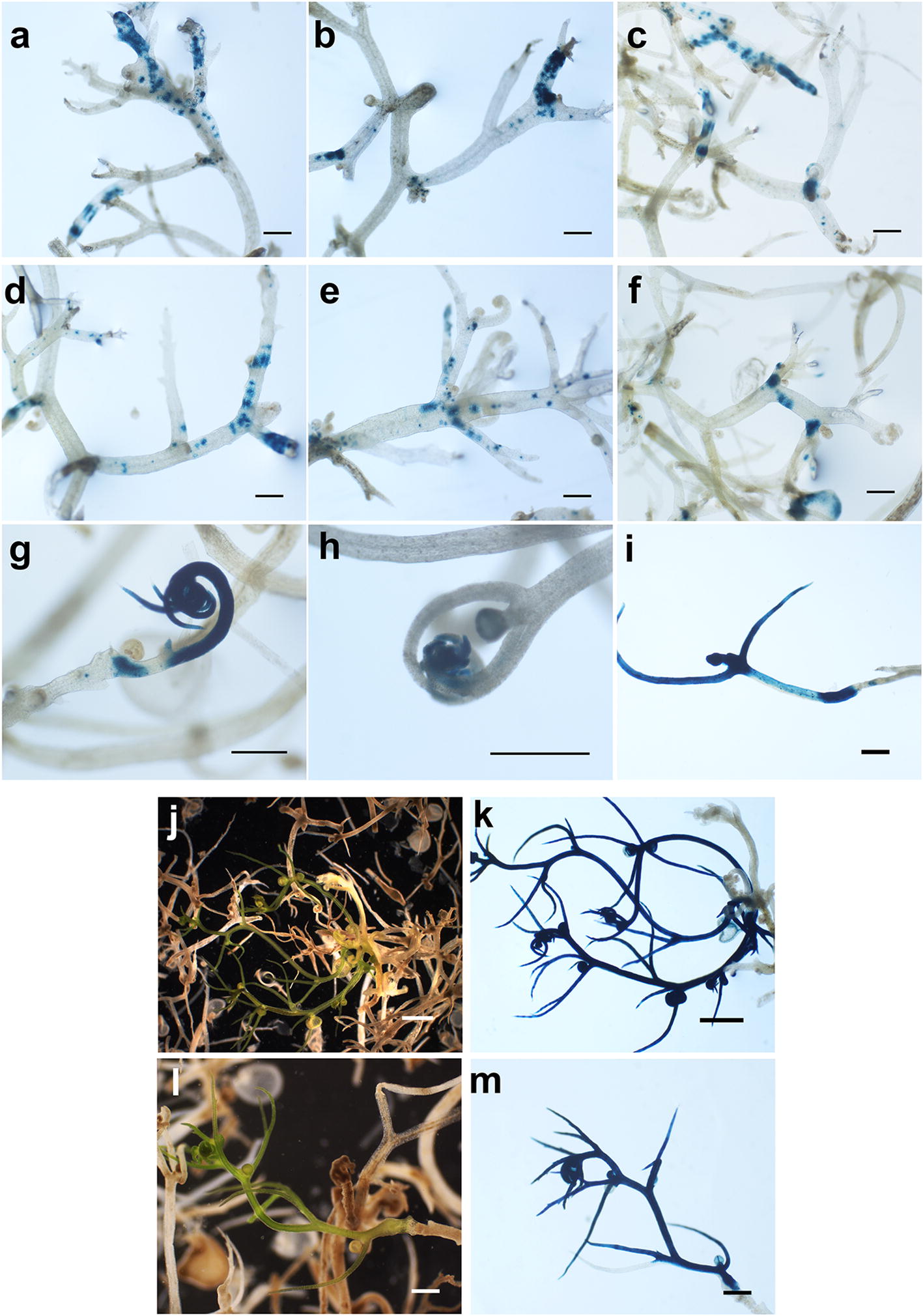
Regeneration and GUS staining kinetics of transformed *U. gibba* plants. **a**–**c** Staining pattern of *U. gibba* tissue transformed with a plasmid carrying p35S-GUS::GFP after 7 days of co-culture with *Agrobacterium*; **d**–**f** GUS staining of *Utricularia* tissue after 18 days of co-culture; **g**, **h** initial transformed buds regenerating after 31 days of co-culture; (i) emerging transformed shoot after 43 days of co-culture; **j**, **l** green shoots growing in selective medium after 45 days of co-culture, with dead, whitish tissue observed around them; **k**, **m** the same shoots stained for GUS activity are shown. Photographs were taken using a SZH10 Olympus microscope. Scale bar for **a**–**i**, **l** and **m** represents 0.5 mm; scale bar for **j**, **k** represents 1 mm

**Fig. 3:**
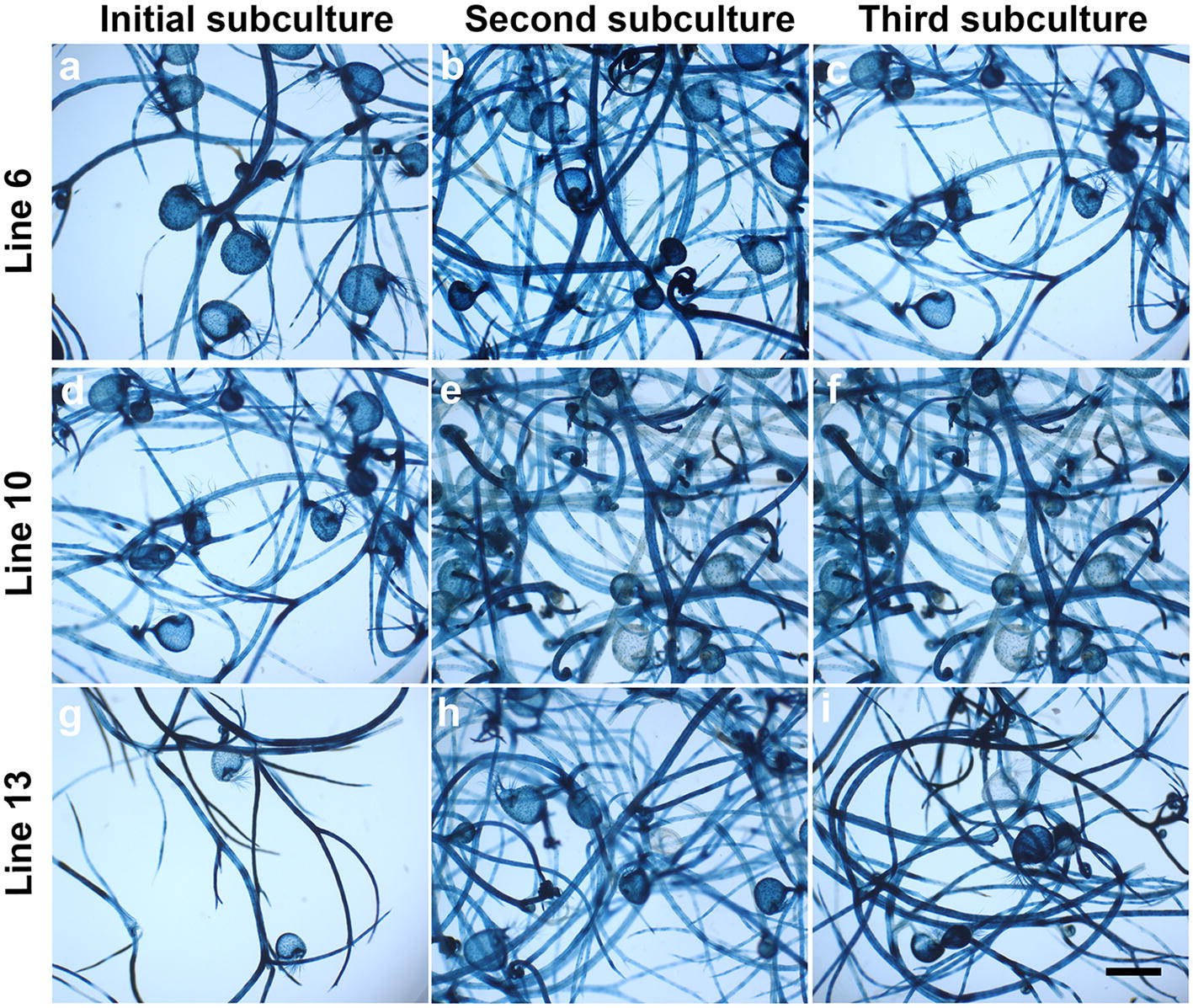
*U. gibba* independent lines sub-cultured in selective medium and histochemical GUS assays. **a**–**c** Transformed p35S-GUS::GFP line 6 showing GUS staining at initial, second and third subculture in selective medium; **d**–**f** GUS activity of line 10 at initial, second and third subculture in selective medium; **g**–**i** GUS expression for line 13 at initial, second and third subculture in selective medium. Photographs were taken using a SZH10 Olympus microscope. Scale bar at bottom right = 1 mm

**Fig. 4:**
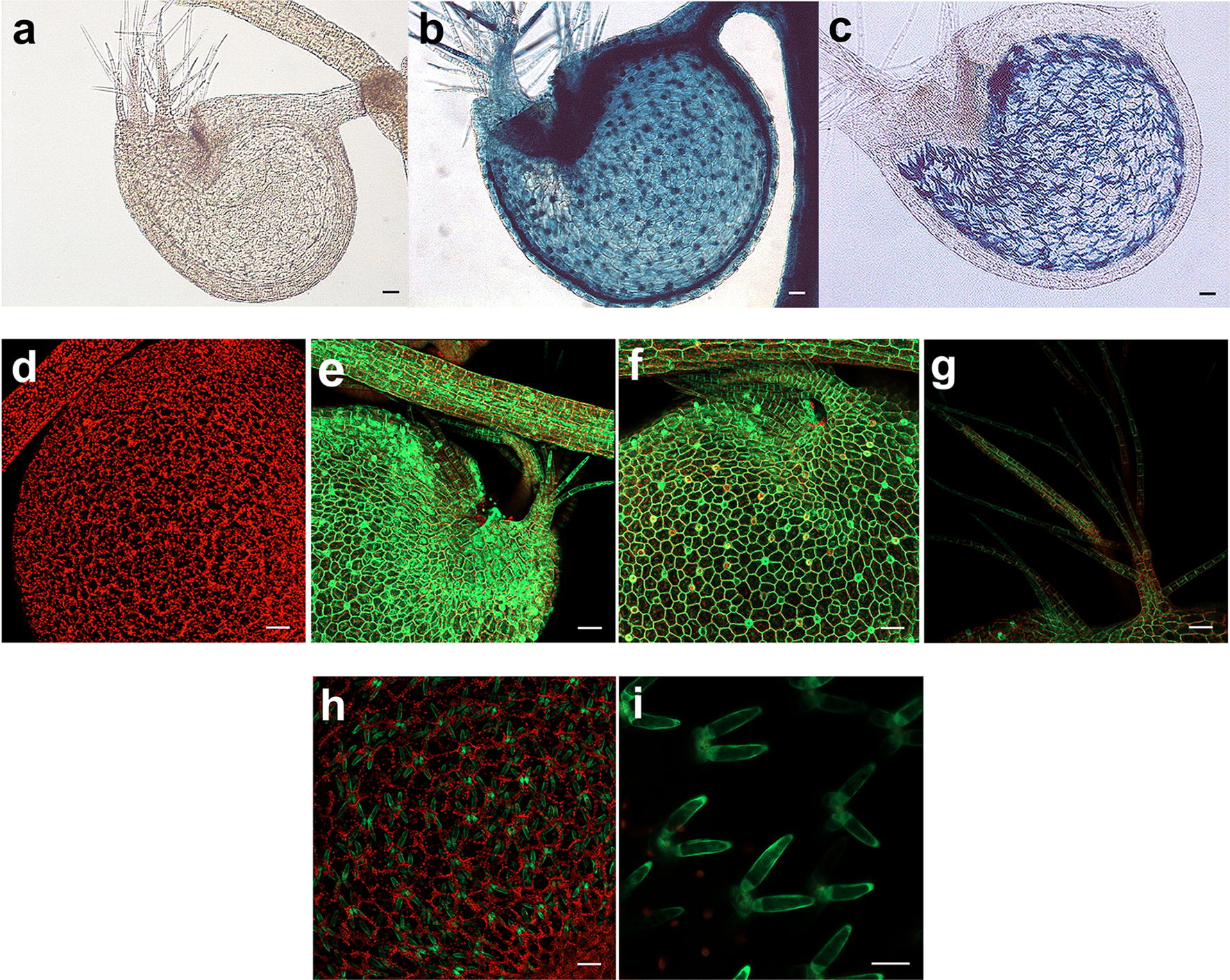
Confocal and Nomarski microscopy *of U. gibba* plants. **a** Wild type trap stained overnight for GUS activity; **b** transformed p35S-GUS::GFP trap, with GUS expression visible in antennae, trap hairs and the globose trap body itself; **c** transformed pRib-GUS::GFP trap, GUS activity is observed in quadrifid cells; **d** wild type trap, no GFP fluorescence is observed; **e**, **f** transformed p35S-GUS::GFP *U. gibba* showing fluorescence throughout bladder traps; **g** close up of antennae and trap hairs of transformed *U. gibba*; **h**, **i** transformed pRib-GUS::GFP trap, where fluorescence is observed only in quadrifid cells. Confocal photographs were taken using a LSM810 Zeiss Microscope and Nomarski photographs using a Leica DRM microscope. Scale bars in **a**–**h**, 50 μM. Bar in **i**, 20 μM

**Fig. 5:**
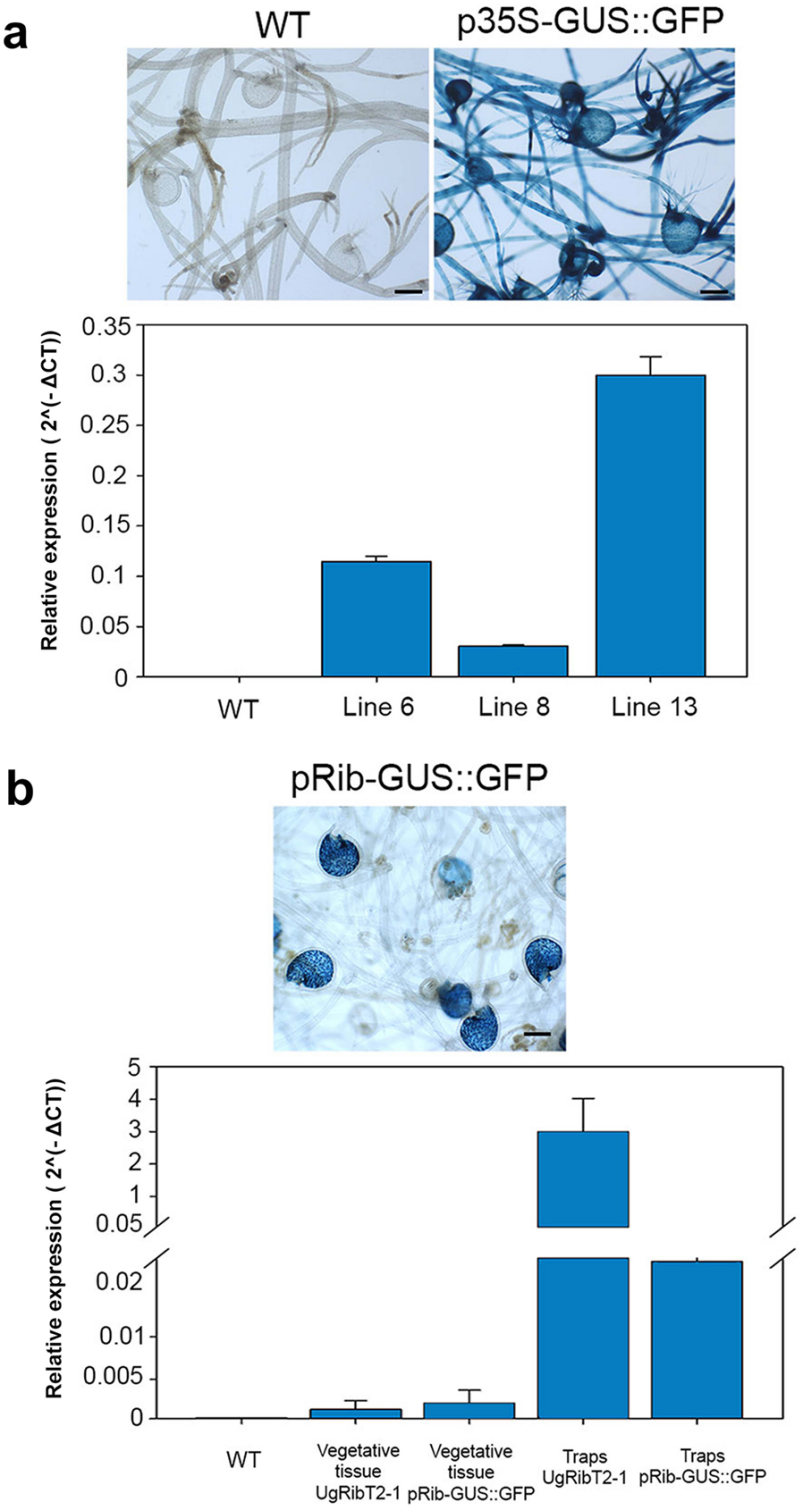
Real Time PCR Analysis of *U. gibba* transgenic lines. **a** Photographs taken using a SZH10 Olympus microscope show *Utricularia* wild type and transgenic p35S-GUS::GFP lines stained overnight for GUS activity. Quantitative reverse transcription PCR analysis for GUS expression of three transgenic lines and one wild type plant are shown. Values are reported as a relative quantification between reporter gene and Ubiquitin gene expressed as 2^(−ΔCT)^. Data are the mean of two biological replicates and three technical replicates for each sample. Standard Error is shown. **b***U. gibba* transgenic plants carrying pRib-GUS::GFP grown for 7 days in media ×0.1 MS, subjected to histochemical GUS assays and photographed using a SZH10 Olympus microscope. Quantitative reverse transcription PCR analysis for GUS and native ribonuclease expression of line pRib-GUS::GFP are shown. A wild type plant as control for GUS expression is also shown. For quantitative analysis, tissue was separated into vegetative and trap tissue. Expression levels are reported as relative expression between reporter or endogenous genes and Ubiquitin gene as 2^(−ΔCT)^. Data are the mean of two biological replicates and three technical replicates for each sample. Standard Error is shown. Scale bars, 1 mm

To better understand how the genetic transformation in *U. gibba* tissue takes place, the transformation process was monitored by GUS staining during the 45 subsequent days after co-culture with *Agrobacterium*. *U. gibba* tissue was sampled from two independent experiments at 7, 18, 31, 43 and 45 days after co-culture with *Agrobacterium*. Each tissue sample was stained with X-Gluc to observe GUS expression foci in co-cultivated tissue, and representative stained tissue was selected for photography. At 7 (Fig. 2a–c) and 18 days after co-culture many small regions showing clear GUS staining were observed in both meristematic and vegetative tissues (Fig. 2d–f). Approximately one month after *Agrobacterium* co-culture, we observed larger sectors with homogeneous GUS staining (Fig. 2g, h). After 6 weeks in selective medium, clearly distinguishable green sectors were observed after staining that showed homogeneous GUS activity (Fig. 2i). Application of BAP did not result in the formation of callus tissues but rather it directly promoted a more rapid de novo formation of meristems that developed in new plant branches that could be dissected to establish a new plant. Between seven and 8 weeks of growth in selective media, green sectors that were clearly visible were dissected and separated from the original dead tissue for further propagation. Initially, we propagated three independent transgenic lines from two independent transformation experiments for further analysis. Putatively transformed segments were stained to corroborate that they expressed the GUS reporter gene. We found that all green segments collected from the initial round of selection and that remained green after subcultivation in media containing PPT were clearly GUS positive (Fig. 2j–m). To confirm that the PPT resistant and GUS positive segments were stably transformed, three independent transformed segments containing p35S-GUS::GFP were sub-cultivated three times for 1 month in fresh media without PPT and then tested for GUS expression. We observed that the three clones maintained the expression of the GUS reporter gene after each of the three subcultures (Fig. 3). In all transgenic lines examined, the 35S promoter was found to directly express in stolons and leaf-like structures, as well as in the entire trap walls including antennae and trap hairs (Fig. 4). No GUS or GFP expression was ever observed in wild type tissue even after long incubation times in histochemical assays (Fig. 4). To confirm that GUS positive clones were indeed transgenic, we quantified the expression level of p35S-GUS::GFP in *U. gibba* tissue by Real Time PCR. Three different lines expressing GUS activity and one wild type line were analyzed. Transgenic lines displayed different levels of expression, as expected from independent lines in which T-DNA is probably inserted in different regions of the *U. gibba* genome (Fig. 5a).

### Plants transformed with the pRibonuclease-GUS::GFP construct show cell-type-specific expression

The development of plant transformation protocols facilitates the functional characterization of genes and their patterns of expression. To test the *U. gibba* transformation system for gene expression research, we analyzed the expression of an organ-specific gene. We previously reported the identification of genes that are specifically expressed in *U. gibba* traps, some of which could be implicated in P uptake from prey digestion [41]. Among the trap-specific genes, we selected one that was very strongly expressed (See Additional file 1: Figure S2). This gene, originally identified as unitig_26.g10301.t1 in the *U. gibba* genome, was renamed UgRibT2-1. It encodes a protein that is a member of the Ribonuclease-related protein family HOM04D000621. This family has a conserved Ribonuclease_T2 protein domain, and is encoded by 5 genes in *A. thaliana*, 7 in *S. lycopersicum* and 3 in *U. gibba* (https://bioinformatics.psb.ugent.be/plaza; see Additional file 1: Figure S2). The 5′-upstream intergenic region of UgRibT2-1 is 1514 nucleotides, which we selected as the promoter region for this trap specific gene (see Additional file 1: Figure S3). To determine the expression patterns directed by the UgRibT2-1 promoter, we created a transcriptional fusion between this promoter and a *UidA* (GUS)-GFP reporter gene, which we named pRib-GUS::GFP. Using the transformation system reported above, we generated 8 independent transgenic *U. gibba* lines harboring the UgRib-GUS-GFP construct. Transgenic UgRib-GUS-GFP plants were subjected to histochemical GUS analysis and confocal microscopy to detect the presence of GFP. We found that expression of the ribonuclease promoter was specific to the trap, more precisely to specialized quadrifid gland cells (Fig. 4c, h, i). These cells are involved in transport, digestion and absorption of prey-derived nutrients inside the trap. Therefore, we propose that UgRibT2-1 has a role in nucleic acid degradation during prey digestion (Fig. 5b).

To quantify the expression level of pRib-GUS::GFP and compare it with UgRibT2-1 in *U. gibba* plants, real time PCR analysis was performed. RNAs extracted from transgenic lines were analyzed after collecting traps and vegetative tissue separately. We found that the level of expression directed by the UgRibT2-1 promoter is over 2000 times higher in traps than in vegetative tissues, whereas pRib-GUS::GFP expressed 10 times greater in traps than vegetative tissue. Although the pRib promoter did direct the expected trap-specific expression pattern, its lower expression strength suggests that regulatory elements of the pRib promoter important to determining expression level are missing in the 1500 bp that we used as pRib promoter. Since this promoter includes all of the 5′ flaking intergenic region of RibT2-1, it is possible that an enhancer element is present inside the transcribed region of this gene (Fig. 5b).

### Detection of *UidA* (GUS) and phosphinothricin acetyl transferase (BAR) in *U. gibba* transgenic lines

To confirm that transgenes were integrated into the *U. gibba* genome, DNA from 5 lines each of p35S-GUS::GFP and pRib-GUS::GFP and 3 plants of wild type were isolated. In transgenic lines, 332pb and 428pb fragments were amplified corresponding to *UidA* (GUS) and BAR amplicons. In wild type lines, these fragments were not observed. As a positive control, a 239 bp fragment was synthesized corresponding to an endogenous Ubiquitine gene, which can be observed in both transgenic and untransformed control lines (Additional file 1: Figure S4).

## Discussion

Gene transfer technology in plants has become an indispensable tool to study plant biology and functional genomics. Transformation protocols have been developed for many plant species, but because of the complexity of tissue culture procedures involved or the lack of reproducibility, many of these protocols are not widely adopted. Therefore, when developing a plant transformation system, one should aim to generate a simple, inexpensive and reproducible transformation system to facilitate broad adoption of the new technology. Here, we report the development of a simple, rapid and reproducible system for genetic transformation of *U. gibba*. Taking advantage of vegetative propagation of this carnivorous plant, production of homogenously transformed plants can be easily achieved. Although the efficiency of the *U. gibba* transformation protocol reported here can likely be further optimized, in less than 3 months, starting with sufficient propagated tissue, several independent transgenic lines of at least 5 to 10 different constructs can be easily produced. By dissecting the original transgenic material obtained by the PPT selection procedure, large amounts of tissue for different assays or gene expression studies can be generated in 1–2 months. Our transformation protocol could in principle be used to generate knock-out mutants using the CRISPR/Cas genome editing system [37].

Using this transformation system, we have shown that the CaMV 35S promoter can be expressed in vegetative tissue of *U. gibba*. We also examined expression directed by the promoter of the UgRibT2-1 gene. This encodes a type T2 ribonuclease that is most probably secreted inside the *U. gibba* traps, as its coding sequences contain a secretion peptide at its N-terminus. We found that expression of pRib-GUS::GFP is specific to quadrifid cells that are located in the inner parts of traps. Quadrifid gland cells have been proposed to play an important role in prey digestion; they secrete digestive enzymes and are also where nutrient uptake takes place [20, 24]. The finding that UgRibT2-1 encodes a ribonuclease potentially involved in hydrolyzing nucleic acids supports the aforementioned notion that quadrifid cells play an important role in prey digestion. Ribonuclease activity in these cells could lead to digestion and subsequent assimilation of phosphorus released from the remains of the prey. In the carnivorous plant *Nepenthes*, RNAse activity was demonstrated when spinach RNA was added to trap fluid [42]. Under these conditions, expression of ribonuclease NvRN1 was demonstrated in pitcher tissues. Activity of hydrolases was also reported in both empty and fed traps of *U. gibba*, suggesting that expression of relevant genes and translation of digestive enzymes is continuous in traps [24]. In tomato, ribonucleases expressed in the root play an important role during phosphate starvation, facilitating scavenging of organic sources of Pi present in the rhizosphere [43]. It is interesting that a ribonuclease expressed in *Arabidopsis* and tomato roots is expressed in the trap of the rootless *U. gibba*, perhaps suggesting that many of the nutrient uptake functions of root systems were re-deployed in the traps of these carnivorous plants. Moreover, analysis of the microbiome of the *U. gibba* traps showed that many bacterial species normally present in the plant rhizosphere are present in *U. gibba* traps [25]. It has been reported that secretory ribonucleases play an important role in phosphate and nitrogen scavenging in the gut of humans and zebrafish [44]. In connection, we hypothesize that the UgRib-T2.1 could have acquired dual functions, as is the case of animals and plants.

*Note* While this work was being prepared for publication another group reported a transformation protocol for *U. gibba* using solid media [45, 46]. Our work uses an alternative system in liquid media that leads to rapid recovery and propagation of transformed plants.

## Conclusions

We developed a simple and rapid method for genetic transformation of *U. gibba* that allows production of transgenic lines in less than 3 months. Genetic transformation of *U. gibba*, together with access to a reference genome and comprehensive transcriptomic data, should facilitate the use of *U. gibba* as a model system to study the complex developmental processes involved in trap development, and to study nutrient uptake in a rootless plant [28, 32]. Additionally, *U. gibba* could be used as a heterologous expression system for expression of orthologous genes of other carnivorous or aquatic plants. Of these plant groups, genomes for *Cephalotus follicularis* and water lily were recently completely sequenced [27, 47].The transformation system of *U. gibba* could be powerful for functional genomics and evolutionary studies in these important plant genomes. One of the most important advantages of the *U. gibba* genome is the fact that it is very compact, but contains the full gene repertoire required for a fully functional, flowering plant.

## Materials and methods

### Plant material and propagation

*Utricularia gibba* seeds were collected near Umécuaro, Michoacán, México. A culture was established from seeds that were disinfected with 70% ethanol for 3 min, thereafter 20% v/v bleach in water for 7 min, followed by sterile water washes. Seeds were then germinated in ¼ MS liquid medium supplemented with 2% sucrose and grown in Percival chambers with 16 h light and 8 h darkness at 22 °C. Once the culture was established, *Utricularia* tissue was sub-cultured to 70 ml of ¼ MS medium in glass bottles of 220 ml of capacity every 4 to 6 weeks in order to maintain culture freshness.

### Selection of candidate gene for *U. gibba* transformation

From RNA-seq data recently reported (Cervantes-Pérez et al., in press), we selected genes with strong and specific expression in the traps. The gene unitig_26.g10301.t1 (CoGe: GeneModels_PacBioAssembly4.gff (vv1.1); Genome: PacBio v1.1 (vPacBio v1.1) gid: 28048) was the chosen candidate because of its high expression in trap tissue transcriptomes. In order to characterize the putative function of this gene, we performed an analysis of gene family conservation in the web-tool PLAZA (https://bioinformatics.psb.ugent.be). Then we made a homology sequence search against Arabidopsis and tomato in CoGe (https://genomevolution.org/CoGe/CoGeBlast.pl) and the Sol Genomics Network (solgenomics.net). Additionally, in the tool GEvo, synteny between these genes was checked (https://genomevolution.org/r/19pyx), and protein domain conservation was checked in HMMER (https://www.ebi.ac.uk) (see Additional file 1: Figure S2). By synteny and homology, we determined this gene to encode an *Utricularia gibba* ribonuclease. Promoter analysis was performed in the MEME suite (http://meme-suite.org/).

### Plasmid constructions

Oligonucleotides were designed to amplify the 35S promoter from vector pFAST G02 (VIB UGENT for Plant Systems Biology) and cloned in pDONR221 (Invitrogen) using a GATEWAY BP enzyme (Invitrogen). The construct was then transferred into the destination vector pBGWFS7 (VIB UGENT for Plant Systems Biology) by GATEWAY LR Kit (Invitrogen) to generate a transcriptional fusion driving GUS-GFP expression (See Additional file 1). Oligonucleotides were designed to amplify the *Utricularia* ribonuclease promoter (See Additional file 1: Table S1). The resulting DNA fragment was first cloned into pDONR221 and then transferred to pBGWFS7 by GATEWAY technology. Both transcriptional fusions p35S-GUS::GFP and pRib-GUS::GFP (the *U. gibba* Ribonuclease promoter) were transformed into *Agrobacterium tumefaciens* by electroporation using BIO-RAD Micropulser equipment and used to obtain *U. gibba* transgenic lines.

### Selective medium

To establish a system of selection for *U. gibba*, MS liquid medium, supplemented with different concentrations of Glufosinate-ammonium PESTANAL (45520 Sigma) 6, 10, 15 and 20 mg/l, were used to test for inhibition of *U. gibba* growth in petri dishes.

### Genetic transformation

To generate tissue for transformation experiments, *U. gibba* was cultivated in 220 ml flasks with 70 ml of liquid MS media for 1 month. Thereafter, tissue was collected and placed on sterile paper towels to remove excess moisture, and fresh weight was determined to calculate the number of transformants per gram of fresh weight of initial material. Approximately 1 g of *U. gibba* tissue was then transferred for 1 week to 250 ml Erlenmeyer flasks with 70 ml of fresh MS liquid medium supplemented with Benzilaminopurine (BAP) 0.25 mg/l. After 1 week in media with BAP, all vegetative mass was collected and cut with a sharp scalpel into approximately 5 mm pieces; about 300 pieces/g of fresh weight tissue. For co-cultivation, 5 ml of *Agrobacterium tumefaciens* strain GPV 2260 culture was grown overnight at OD _600_ = 1, centrifuged, washed with MS medium and resuspended into 0.5 ml of MS media. Fragments of *U. gibba* tissue were returned into 250 ml flasks with 70 ml of fresh MS media and inoculated with 0.5 ml of concentrated *Agrobacterium* suspension for co-culture. Acetosiringone (100 µM), glutamic acid (300 mg/l) and casamino acids (1 g/L) were also added for proper bacterial growth. After 3 days in an orbital shaker, the *Utricularia*-*Agrobacterium* co-culture was washed with sterile water and then distributed among four 90 mm × 25 mm Petri dishes with 24 ml of selection MS liquid medium supplemented with BAP (0.25 mg/l), glufosinate ammonium (10 mg/l) and cefatoxime (250 mg/l) to allow bacterial control. Two weeks after co-culture, tissue was subcultured in MS medium with PPT, cefatoxime and without BAP in order to allow elongation of primordial shoots that formed. Regenerated transgenic tissue was subcultivated every 15 days in selective MS liquid medium three additional times.

### Histochemical GUS assay

Transgenic and nontransgenic *U. gibba* tissues were incubated in GUS reaction buffer (0.5 mg/mL of 5‐bromo‐4‐chloro‐3‐indolyl‐b‐d‐glucuronide in 100 mM sodium phosphate, pH 7.0) overnight at 37 °C. The tissues were cleared by the Malamy and Benfey method [48] and observed and photographed using Nomarski optics in a Leica DMR microscope and a SZH10 Olympus stereomicroscope.

### Utricularia DNA isolation and PCR analysis

Tissues from *U. gibba* wild type and transgenic lines carrying the p35S-GUS::GFP and pRib-GUS::GFP were collected, frozen in liquid nitrogen and ground to isolate total DNA using the DNeasy Power Plant Pro Kit from Qiagen. 5 ng of DNA from each sample was used for PCR reactions in a Veriti instrument from Applied Biosystems. The PCR amplification conditions were as follows: for the GUS gene, 1:30 min at 94 °C, and 35 cycles at 94 °C for 30 s, 60 °C for 20 s and 72 °C for 30 s. For the BAR gene and Ubiquitin genes, 1:30 min at 94 °C, and 35 cycles at 94 °C for 30 s, 65 °C for 20 s and 72 °C for 25 s.

### Real time quantitative reverse transcription PCR analysis

*Utricularia gibba* wild type and transformed plants carrying the p35S-GUS::GFP were sub-cultured in ¼ MS medium for a week in order to obtain a fresh culture growing in optimal conditions. Then tissue was collected, frozen and ground to isolate total RNA using Trizol Reagent (Life Technologies). *U. gibba* plants transformed with pRib-GUS::GFP were grown in medium 0.1× MS for a week, so that the culture was fresh. Afterward, vegetative tissue and traps were collected separately, frozen and ground as previously described. For RNA isolation from trap tissue, the Direct-zol RNA miniprep from Zymo Research kit was used. 500 ng of each RNA was used to carry out the reverse transcription reaction with SuperScript III reverse transcriptase (Invitrogen), following the manufacturer’s protocol. The qPCR was performed in MIC (Magnetic Induction Cycler from Biomolecular Systems) equipment using specific primers (See Additional file 1) and the reagent SensiFast TM SYBR No-ROX Kit from Bioline. The PCR conditions were 95 °C for 2 min, followed by 40 cycles of 95 °C for 5 s, 65 °C for 10 s and 72 °C for 20 s. The abundances of the GUS gene and UgRibT2-1 were calculated relative to the Ubiquitin gene for each sample.

## Supplementary information

**Additional file 1: Figure S1.** Selection markers for *U. gibba* in vitro culture. *Utricularia* tissue exposed to different concentrations and selection markers, observed and photographed at 9 day in MS culture. Tissue placed in Glufosinate-ammonium PESTANAL at 6,10,15 and 20 mg/l showed bleaching and dead tissue in all treatments. *Utricularia* tissue subjected to Kanamycin showed no effect in viability, and green tissue was observed at 20, 40, 60 and 80 mg/l. At low Hygromycin concentrations (5, 10 and 20 mg/l), *U. gibba* remained green and alive, while at 30 mg/l, dead tissue was observed. Wild type *Utricularia* photographs as experimental controls are shown. Scale bar, 1 cm. **Figure S2.** Ribonuclease gene of *U. gibba*. a) Conserved protein domain structure among putative orthologs of Ribonuclease T2 genes in Arabidopsis, tomato and *U. gibba*. The graphic represents the conserved Ribonuclease_T2 domain in the three proteins. b) Expression levels of the *U. gibba* Ribonuclease gene (unitig_26.g10301.t1) in 10 RNA-Seq vegetative tissue and one trap libraries. On the Y axis we show transcripts per million for each library and on the X axis each condition is shown. **Figure S3.** In silico promoter analysis of Ribonuclease T2 genes. For each analysis the name of promoter region, confidence level for identifications and motif locations along the region are represented. Colored blocks indicate DNA motif type identified in the MEME software suite. **Figure S4.** DNA of five independent transgenic p35S-GUS::GFP and pRib-GUS::GFP lines and 3 independent WT lines was isolated and PCR reaction performed. A 332 bp fragment for the UidA gene and 428 bp for the BAR gene of transformed lines are shown. A 239 bp fragment for Ubiquitin gene as the control in transgenic and non-transgenic lines is also shown. The GeneRuler 1 Kb Plus DNA Ladder (Thermo Scientific) was used. **Table S1.** Oligonucleotides sequence.

## Data Availability

Data sharing not applicable to this article, as no datasets were generated in the current study.

## Abbreviations

BAP: Benzilaminopurine
PPT: Phosphinothricin
