# Supplementary material for: Agrobacterium tumefaciens mediated transformation of the aquatic carnivorous plant Utricularia gibba
Source: Plant Methods. 2020 Apr 10;16:50. doi: 10.1186/s13007-020-00592-7 (PMC7149871; doi:10.1186/s13007-020-00592-7)
Supplement: Supplementary file 1 — Additional file 1: Figure S1. Selection markers for U. gibba in vitro culture. Utricularia tissue exposed to different concentrations and selection markers, observed and photographed at 9 day in MS culture. Tissue placed in Glufosinate-ammonium PESTANAL at 6,10,15 and 20 mg/l showed bleaching and dead tissue in all treatments. Utricularia tissue subjected to Kanamycin showed no effect in viability, and green tissue was observed at 20, 40, 60 and 80 mg/l. At low Hygromycin concentrations (5, 10 and 20 mg/l), U. gibba remained green and alive, while at 30 mg/l, dead tissue was observed. Wild type Utricularia photographs as experimental controls are shown. Scale bar, 1 cm. Figure S2. Ribonuclease gene of U. gibba. a) Conserved protein domain structure among putative orthologs of Ribonuclease T2 genes in Arabidopsis, tomato and U. gibba. The graphic represents the conserved Ribonuclease_T2 domain in the three proteins. b) Expression levels of the U. gibba Ribonuclease gene (unitig_26.g10301.t1) in 10 RNA-Seq vegetative tissue and one trap libraries. On the Y axis we show transcripts per million for each library and on the X axis each condition is shown. Figure S3. In silico promoter analysis of Ribonuclease T2 genes. For each analysis the name of promoter region, confidence level for identifications and motif locations along the region are represented. Colored blocks indicate DNA motif type identified in the MEME software suite. Figure S4. DNA of five independent transgenic p35S-GUS::GFP and pRib-GUS::GFP lines and 3 independent WT lines was isolated and PCR reaction performed. A 332 bp fragment for the UidA gene and 428 bp for the BAR gene of transformed lines are shown. A 239 bp fragment for Ubiquitin gene as the control in transgenic and non-transgenic lines is also shown. The GeneRuler 1 Kb Plus DNA Ladder (Thermo Scientific) was used. Table S1. Oligonucleotides sequence. [file 13007_2020_592_MOESM1_ESM.pdf]

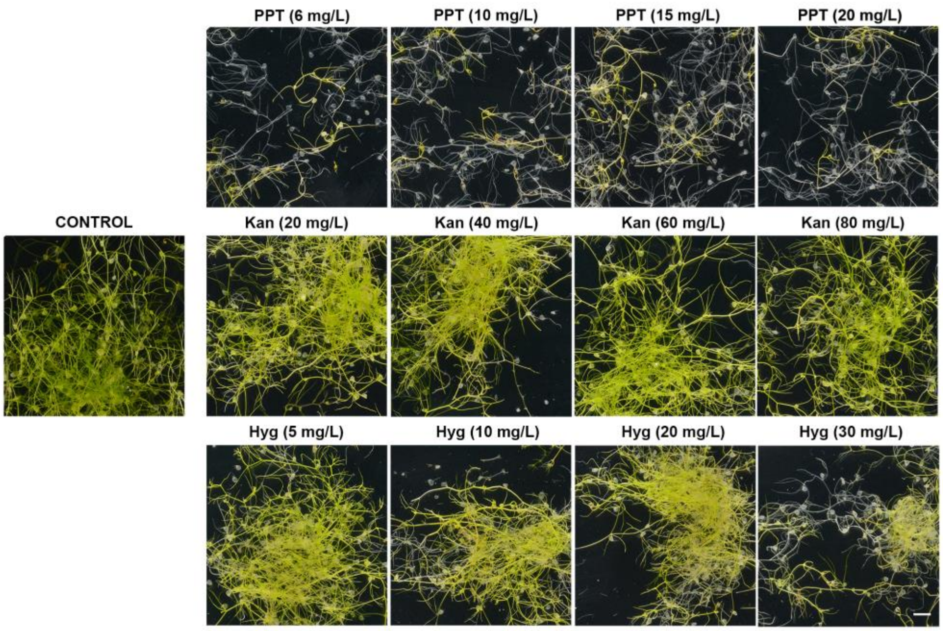

Figure S1

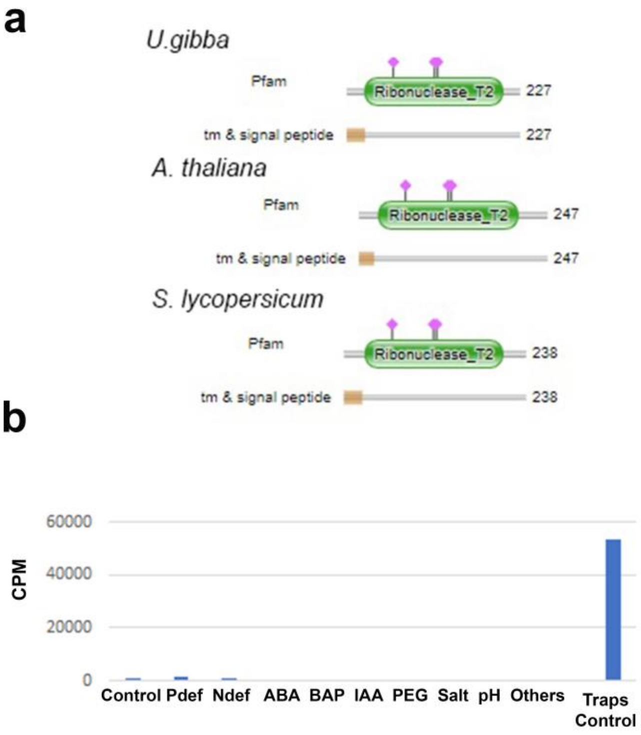

Figure S2

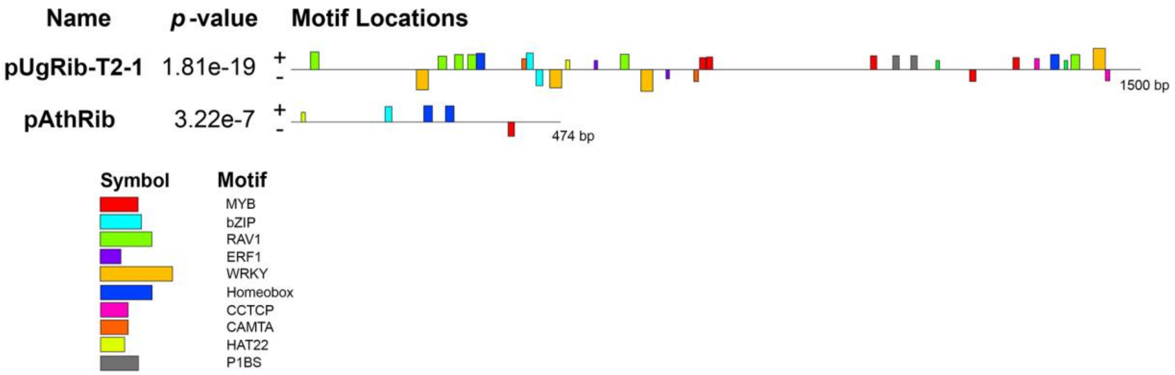

Figure S3

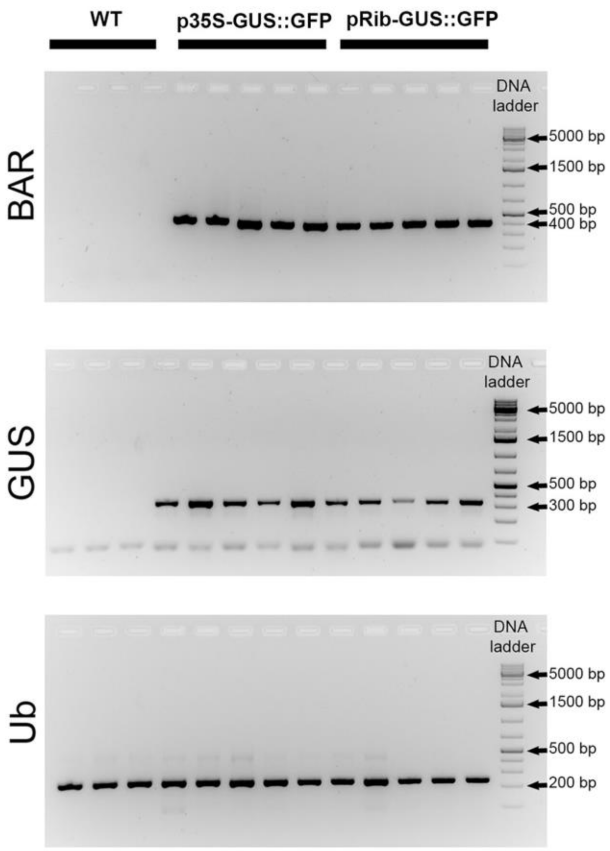

Figure S4

Table S1

OLIGONUCLEOTIDE SEQUENCES

GUS Fwd	5’ TGCTGTCGGCTTTAACCTCT 3’
GUS Rev	5’ GGCACAGCACATCAAAGAGA 3’
BAR Fwd	5’ ACTACATCGAGACAAGCACGGTC 3’
BAR Rev	5’ GTTTCTGGCAGCTGGACTTCAGC 3’
Ubiquitine Fwd	5’ GAGGTTGAAAGCTCGGACACTATC 3’
Ubiquitine Rev	5’ CTCTCGACCTCTAGAGTGATGGTT 3’
p35S Fwd	5’ GGGGACAAGTTTGTACAAAAAAGCAGGCTACACTAGAGCCAAGCTGATCTC 3’
p35S Rev	5’ GGGGACCACTTTGTACAAGAAAGCTGGGTATCGACTAGAATAGTAAATTG 3’
pRib Fwd	5’GGGGACAAGTTTGTACAAAAAAGCAGGCTACGAGATTCATATTCGCCACACACTG 3’
pRib Rev	5’ GGGGACCACTTTGTACAAGAAAGCTGGGTAAGGGAAGAAGATGACATGTTTATTC 3’
Rib Fwd	5’ AGCTCTCGCGCAAGCAGGAATCCA 3’
Rib Rev	5’ CTCGAACAATTGCTCGTCGCCGTA 3’
